# Supplementary material for: Critical success factors influencing business intelligence adoption: Evidence from Yemen
Source: PLoS One. 2026 Feb 25;21(2):e0343217. doi: 10.1371/journal.pone.0343217 (PMC12935231; doi:10.1371/journal.pone.0343217)
Supplement: S1 Appendix — This file contains the detailed steps of the Rough SWARA method, including expert initial preferences, calculated rough boundaries, and final weight derivations. (DOCX) [file pone.0343217.s001.docx]

# **S1 Appendix**

## **Table A1. Individual expert rankings of the sixteen CSFs (E1–E12).**

| E/C | C1 | C2 | C3 | C4 | C5 | C6 | C7 | C8 | C9 | C10 | C11 | C12 | C13 | C14 | C15 | C16 |
| --- | --- | --- | --- | --- | --- | --- | --- | --- | --- | --- | --- | --- | --- | --- | --- | --- |
| E1 | 8 | 12 | 3 | 16 | 5 | 15 | 7 | 11 | 14 | 4 | 10 | 6 | 13 | 1 | 9 | 2 |
| E2 | 1 | 13 | 5 | 6 | 10 | 15 | 16 | 9 | 12 | 4 | 7 | 11 | 2 | 3 | 8 | 14 |
| E3 | 10 | 14 | 11 | 6 | 9 | 1 | 12 | 15 | 16 | 3 | 7 | 4 | 5 | 2 | 13 | 8 |
| E4 | 7 | 8 | 3 | 5 | 6 | 16 | 1 | 15 | 12 | 9 | 10 | 14 | 4 | 11 | 13 | 2 |
| E5 | 10 | 11 | 12 | 14 | 15 | 9 | 8 | 1 | 6 | 7 | 5 | 13 | 3 | 2 | 16 | 4 |
| E6 | 4 | 15 | 16 | 2 | 6 | 3 | 10 | 5 | 12 | 13 | 14 | 7 | 9 | 1 | 8 | 11 |
| E7 | 2 | 8 | 1 | 15 | 5 | 12 | 10 | 6 | 9 | 3 | 11 | 13 | 7 | 16 | 14 | 4 |
| E8 | 9 | 11 | 6 | 16 | 13 | 3 | 8 | 14 | 15 | 5 | 2 | 10 | 1 | 7 | 4 | 12 |
| E9 | 6 | 10 | 9 | 1 | 12 | 4 | 2 | 8 | 5 | 3 | 11 | 16 | 13 | 14 | 15 | 7 |
| E10 | 2 | 1 | 3 | 13 | 7 | 5 | 12 | 15 | 10 | 8 | 6 | 9 | 11 | 14 | 16 | 4 |
| E11 | 8 | 2 | 16 | 13 | 9 | 11 | 7 | 3 | 1 | 14 | 5 | 4 | 6 | 12 | 10 | 15 |
| E12 | 12 | 9 | 13 | 15 | 16 | 7 | 10 | 5 | 3 | 8 | 6 | 2 | 4 | 14 | 11 | 1 |

## **Table A2. Rough Group Matrix 𝑅𝑁 (𝐶𝑗) of CSFs calculated by equation (1).**

| **Rough Matrix 𝑅𝑁 (𝐶𝑗)** | $\left[ \boldsymbol{c}_{\boldsymbol{j}}^{\boldsymbol{L}} , \boldsymbol{c}_{\boldsymbol{j}}^{\boldsymbol{U}} \right]$ |
| --- | --- |
| RN(c13) | [3.63, 9.70] |
| RN(c1) | [3.89, 9.12] |
| RN(c10) | [4.32, 9.63] |
| RN(c16) | [3.65, 10.65] |
| RN(c11) | [5.48, 10.27] |
| RN(c14) | [4.03, 12.18] |
| RN(c3) | [4.44, 12.10] |
| RN(c6) | [4.61, 12.37] |
| RN(c7) | [5.62, 11.42] |
| RN(c8) | [5.30, 12.51] |
| RN(c12) | [5.71, 12.39] |
| RN(c5) | [6.79, 12.35] |
| RN(c2) | [6.16, 12.37] |
| RN(c9) | [5.88, 12.94] |
| RN(c4) | [6.01, 13.81] |
| RN(c15) | [8.59, 14.00] |

## **Table A3. Rough Matrix 𝑅𝑁 (𝑆𝑗) of CSFs calculated by equation (2) – (4).**

| **Rough Matrix 𝑅𝑁 (𝑆𝑗)** | $\left[ \boldsymbol{s}_{\boldsymbol{j}}^{\boldsymbol{L}} , \boldsymbol{s}_{\boldsymbol{j}}^{\boldsymbol{U}} \right]$ |
| --- | --- |
| RN(s13) | [1.00, 1.00] |
| RN(s1) | [0.28, 1.06] |
| RN(s10) | [0.31, 1.12] |
| RN(s16) | [0.26, 1.24] |
| RN(s11) | [0.39, 1.20] |
| RN(s14) | [0.29, 1.42] |
| RN(s3) | [0.32, 1.41] |
| RN(s6) | [0.33, 1.44] |
| RN(s7) | [0.40, 1.33] |
| RN(s8) | [0.38, 1.46] |
| RN(s12) | [0.41, 1.44] |
| RN(s5) | [0.48, 1.44] |
| RN(s2) | [0.44, 1.44] |
| RN(s9) | [0.42, 1.51] |
| RN(s4) | [0.43, 1.61] |
| RN(s15) | [0.61, 1.63] |

## **Table A4. Rough Matrix 𝑅𝑁 (𝐾𝑗) of CSFs calculated by equation (6).**

| **Rough Matrix 𝑅𝑁 (𝐾𝑗)** | $\left[ \boldsymbol{k}_{\boldsymbol{j}}^{\boldsymbol{L}}\mathbf{,}\boldsymbol{k}_{\boldsymbol{j}}^{\boldsymbol{U}} \right]$ |
| --- | --- |
| RN(k13) | [1.00, 1.00] |
| RN(k1) | [1.28, 2.06] |
| RN(k10) | [1.31, 2.12] |
| RN(k16) | [1.26, 2.24] |
| RN(k11) | [1.39, 2.20] |
| RN(k14) | [1.29, 2.42] |
| RN(k3) | [1.32, 2.41] |
| RN(k6) | [1.33, 2.44] |
| RN(k7) | [1.40, 2.33] |
| RN(k8) | [1.38, 2.46] |
| RN(k12) | [1.41, 2.44] |
| RN(k5) | [1.48, 2.44] |
| RN(k2) | [1.44, 2.44] |
| RN(k9) | [1.42, 2.51] |
| RN(k4) | [1.43, 2.61] |
| RN(k15) | [1.61, 2.63] |

## **Table A5. Rough Matrix 𝑅𝑁 (𝑄𝑗) of CSFs calculated by equation (8).**

| **Rough Matrix 𝑅𝑁 (𝑄𝑗)** | $\left[ \boldsymbol{q}_{\boldsymbol{j}}^{\boldsymbol{L}}\mathbf{,}\boldsymbol{q}_{\boldsymbol{j}}^{\boldsymbol{U}} \right]$ |
| --- | --- |
| RN(q13) | [1.00, 1.00] |
| RN(q1) | [0.484910, 0.78] |
| RN(q10) | [0.228616, 0.60] |
| RN(q16) | [0.102071, 0.47] |
| RN(q11) | [0.046494, 0.34] |
| RN(q14) | [0.019229, 0.26] |
| RN(q3) | [0.007983, 0.20] |
| RN(q6) | [0.003272, 0.15] |
| RN(q7) | [0.001404, 0.11] |
| RN(q8) | [0.000572, 0.08] |
| RN(q12) | [0.000234, 0.06] |
| RN(q5) | [0.000096, 0.04] |
| RN(q2) | [0.000039, 0.03] |
| RN(q9) | [0.000016, 0.02] |
| RN(q4) | [0.000006, 0.01] |
| RN(q15) | [0.000002, 0.01] |

**Note:** These supplementary materials ensure full transparency and replicability of the results while keeping the main article focused on the prioritized findings and their implications.
