# Supplementary material for: Critical success factors influencing business intelligence adoption: Evidence from Yemen
Source: PLoS One. 2026 Feb 25;21(2):e0343217. doi: 10.1371/journal.pone.0343217 (PMC12935231; doi:10.1371/journal.pone.0343217)
Supplement: S2 Appendix — The survey instrument was used to collect expert judgment. (DOCX) [file pone.0343217.s002.docx]

**First part: Demographic features**

| **1. Company /Sector ...................** |  |  |  |  |
| --- | --- | --- | --- | --- |
| **2. Age** |  |  |  |  |
| o **More than 50** | o **41-50** | o | **30-40** | o **Less than 30** |
| **3. Gender** |  |  |  |  |
|  |  | o **Male** | o **Female** |  |
| **4. Qualification** |  |  |  |  |
| o **PHD MSc** o  **BSc**  o | | |  | o **Diploma** |
| **5. Experience Years ...................** | | | | |
| **6. Position ...................** | | | | |

**The second part: the questions**

**Rank the following critical success factors according to their importance in BI adoption**

****Drag and drop the boxes to rank items***

| **No.** | **The Critical Success Factors** |
| --- | --- |
| **1** | **Organization size:** organization's size in terms of staff and resources/budget. |
| **2** | **IT infrastructure:** Tangible IT resources that provide a foundation to enable present and future business. |
| **3** | **Champion & Balance team Composition:** Consist of a champion(hero/leader) with outstanding experience, and a professional team guided by high-efficiency external consultants. |
| **4** | **Clear vision and business strategic Alignment:** BI vision is clear and aligned to the business strategic vision. |
| **5** | **Regulation:** set of governmental policies influencing technology diffusion. |
| **6** | **Adequate resources:** The amount of technical, financial, and human resources that are allocated to the implementation process. |
| **7** | **Compatibility:** The fitness of BI solution with the existing needs, practices, past experiences, and values of an organization. |
| **8** | **System integration:** The ability of the BI to interact and communicate with other existing systems and databases. |
| **9** | **Data quality:** Data accuracy, comprehensiveness, consistency, and completeness. |
| **10** | **Competitive Pressure:** Is the motivating force for companies to search for innovation to survive in a rapidly changing market. |
| **11** | **Relative advantages:** Expected benefits from BI adoption. |
| **12** | **Complexity:** The perceived difficulty of using the BI. |
| **13** | **Top Management support:** Providing overall direction and support (resources, funding, human skills, and other requirements) from management. |
| **14** | **Information sharing culture:** The values, beliefs, and social ideals shared by organization's employees |
| **15** | **Business driven, scalable & flexible technical framework:** System quality. |
| **16** | **Change management:** managing the changes in functions, processes, or transactions during the adoption process. |
