# Supplementary material for: Critical success factors influencing business intelligence adoption: Evidence from Yemen
Source: PLoS One. 2026 Feb 25;21(2):e0343217. doi: 10.1371/journal.pone.0343217 (PMC12935231; doi:10.1371/journal.pone.0343217)
Supplement: Attachment — Submitted filename: Response_to_Reviewers.docx [file pone.0343217.s004.docx]

# Response to Reviewers

**Manuscript ID: PONE-D-25-33603**
Title: **Critical Success Factors Influencing Business Intelligence Adoption: Evidence from Yemen**
We sincerely thank the Academic Editor and the reviewers for their thoughtful comments and constructive feedback. Below we provide a detailed, point-by-point response in table format. Revisions are tracked in the file 'Revised Manuscript with Track Changes' and a clean version has also been uploaded.

| Reviewer Comment | Author Response | Location of Revision |
| --- | --- | --- |
| Journal Requirement 1 – Style & Formatting: Ensure manuscript follows PLOS ONE style. | Reformatted manuscript, checked against PLOS ONE templates. | Entire manuscript |
| Journal Requirement 2 – Ethics: Clarify participant consent type and process. | Revised Ethics Statement stated: "All participants were adult professionals and provided informed written consent prior to participation. Participants were informed that the study was conducted solely for academic purposes, with participation being voluntary and subject to withdrawal at any time without consequence. They were further assured that their responses would remain fully anonymous and confidential." | Methods section 4.2 – data collection page no. 13 |
| Journal Requirement 3 – Data Availability: Confirm minimal dataset. | We added new section: Data Availability Statement:  All relevant data are within the manuscript. The minimal dataset, including the expert rankings, R-SWARA inputs, calculations, outputs, and the values underlying the tables and figures, has been provided inside the manuscript and the appendix. This data is sufficient to replicate all analyses and findings reported in this paper74. | Data Availability Statement section after conclusion page no. 23 |
| Journal Requirement 4 – Data Availability Statement of the Submission | To address the concern regarding the data sharing plan:  **Data Location:** We confirm that no separate data files or public repository submissions are required. All necessary underlying data for this study—specifically the expert ratings, all R-SWARA calculation data, and sensitivity analysis—are provided in their entirety and in detail within the main manuscript file and in Appendix 1.  **Compliance:** As the data is an integral part of the article's appendix, it will be freely and openly accessible to all readers immediately upon publication, fully satisfying the open data requirement without the need for a separate deposition process.  **Submission Update:** We have now updated the Data Availability Statement within the submission form to reflect this precise location and confirm immediate open access upon acceptance. | Data Availability Statement Page no. 22; Appendix 1 |
| Reviewer #1 #2: The manuscript must describe a technically sound piece of scientific research with data that supports the conclusions. Experiments must be conducted rigorously, with appropriate controls, replication, and sample sizes. The conclusions must be drawn appropriately based on the data presented. Reviewer #1: Partly Reviewer #2: No | Strengthen the demonstration of scientific rigorous to explicitly justify its soundness: **1. Methodological Rigor & Controls:** The R-SWARA method was selected for its specific subjectivity and uncertainty  in handling expert judgment.  **2. Sample Size (Expert Panel):** The sample size of 12 experts is appropriate and well-justified for an MCDM study. In MCDM research, the focus is on the depth of knowledge of a carefully selected expert panel rather than a large, statistical sample. Our panel (detailed in Table 2) was purposively selected to include professionals with extensive, hands-on experience in BI from a diverse range of key sectors in Yemen, ensuring the data is of high quality and contextually relevant.  **3. Replication:** To address replication, we have now included a comprehensive Sensitivity Analysis (Section 5.2). This analysis systematically tests the stability of our results by varying the weight of the top-ranked factor, demonstrating that the overall ranking of CSFs remains robust. This acts as a form of computational replication, confirming the reliability of our findings. **4. Data Supporting Conclusions:** The Discussion (Section 6) has been substantially revised to meticulously link each key finding (e.g., the high rank of Competitive Pressure, the low rank of Top Management Support) directly back to the results data (Table 3) and to existing literature. We explicitly explain these findings in the unique context of Yemen's developing economy, ensuring all conclusions are firmly grounded in and justified by the data presented. | • **Section 4.1 (R-SWARA Method):** Enhanced justification for method selection and rigor. • **Section 4.2 (Data Collection):** Strengthened rationale for expert panel composition. • **Section 5.2 (Sensitivity Analysis):** New section added to demonstrate robustness and replication. • **Section 6 (Discussion):** Entirely restructured to create a clear, logical line from data to interpretation to conclusion, with specific contextual explanations. |
| Reviewer #1: Paper has potential but needs improvement in language, methodological rigor, and contextual interpretation. | We have undertaken a comprehensive revision of the manuscript's language and structure. This included:  - Editing to enhance clarity and reduce wordiness.  - Restructuring of the methodology section to provide clearer justification for R-SWARA selection and robustness tests.  - Strengthened contextual interpretation in the discussion by linking findings to Yemen's specific socio-economic context. | Sections  (Methodology), (Analysis and Results), (Discussion)  pages no. 10, 13, 17 |
| Reviewer #2 – 1: Abstract & Introduction: Too general, lacks research gap and contribution. | Rewritten to emphasize:  - Addresses the empirical research gap in developing economies by investigating Critical Success Factors (CSFs) for BI adoption specifically in Yemen.  - Developing a novel integrated TOEP framework (TOE + Yeoh & Koronios model) and applies the rigorous R-SWARA method.  - Providing actionable, contextualized findings, identifying competitive pressure, data quality, clear vision, and change management as key drivers, while highlighting information sharing culture and system integration as unique challenges in the Yemeni context, which contradicts patterns in stable economies. | Abstract; Section 1 (Introduction) pages no. 1, 2 |
| Reviewer #2 – 2: Literature Review: Descriptive, not analytical; lacks synthesis. | Reorganized into analytical synthesis:  **1. Shift from Description to Analysis**  The review avoids merely stating what previous authors found. Instead, it analyzes the limitations and strengths of the foundational models:  **- Analysis of TOE:** Acknowledging the framework's "comprehensive, holistic, and flexible perspective" (strength) but immediately pivots to its "generality" as a major limitation for a "complex, context-specific system like BI," noting its lack of specificity for process factors (weakness).  **- Analysis of Yeoh and Koronios Model:** Praising its focus on BI's "unique complexities" (strength) but highlights its "major theoretical shortfall": the neglect of the broader environmental context (weakness).  **2. Achieving Synthesis and Justification**  Synthesis means blending elements to form a new, coherent argument.  **- Synthesis of Gaps:** The text explicitly synthesizes the weaknesses of the two models, arguing that the TOE is too generic (missing process detail) while the Yeoh and Koronios model is too internally focused (missing environmental detail).  **- Logical Framework Justification:** This synthesis leads directly and logically to the study's primary theoretical contribution: the new Technology-Organization-Environment-Process (TOEP) framework. The TOEP model is presented not as a random addition, but as the necessary solution to the combined theoretical shortcomings identified.  **3. Clear Research Gap Statement**  The argument concludes by firmly grounding the theoretical gap in the research context:  - The need for the TOEP framework is tied to the complexity of the Yemeni context, where both "external pressures (environment) and internal implementation (process) are critical.  - The review ends by stating that the integrated framework and methodology will "effectively investigate the CSFs for BI adoption in the under-researched and challenging context of Yemen." | Section 2 (Literature Review & Background) pages no. 2 - 5 |
| Reviewer #2 – 3: Methodology: Lacks justification for weighting and robustness tests. | -Expanded methodology section with detailed justification for R-SWARA selection over AHP/FAHP, highlighting its advantages in handling expert judgment uncertainty through rough numbers  - Added sensitivity analysis (Section 5.2, Table 9) testing robustness by varying the top factor's weight from 0.1 to 0.9, confirming ranking stability  - Enhanced description of sample size and sexpert selection criteria and profile diversity (Table 2) | Section 4.1 (pages 10)  Section 5.2 (pages 16-17) |
| Reviewer #2 – 4: Results: Hard to follow; large tables dominate; little novelty. | **- Improved Readability:** Condensed large tables: Shifted detailed tables (e.g., raw expert rankings, R-SWARA calculations) to Appendix A, keeping only the essential summary tables in the main body. The "Results" section in the main paper (Section 5) becomes much less cluttered and easier to follow. The core findings are now presented more clearly.  **- Enhanced Presentation/Added Visualization:** Incorporated graphs and figures (e.g., Figure 4: CSF Prioritization, Figure 5: Sensitivity Analysis) to improve visual comprehension and provide an immediate, visual understanding of the CSF rankings and the stability of the results, directly combating the "hard to follow" critique.  **- Emphasized Novelty:** Emphasized the Yemeni-context-specific insights in the Discussion and Conclusion sections to underscore the study's unique contribution. The discussion (Section 6) explicitly contrasts their findings from Yemen with established literature. Key contextual insights include competitive pressure as the top driver, reflecting a survivalist market. Top Management Support and Relative Advantages ranked surprisingly low, suggesting a lack of awareness or realization of BI's value. Information culture and system integration are identified not as drivers but as fundamental barriers in the Yemeni context, a significant theoretical insight. | Section 5 (Analysis & Results); Appendix A |
| Reviewer #2 – 5: Discussion: Weak theoretical linkage and vague policy implications. | **- Enhanced Theoretical Foundation: By** deliberately weaving the findings back to established theories, the authors have elevated the discussion from a mere presentation of results to a meaningful scholarly interpretation.  **- Actionable Implications:** The paper now clearly separates guidance for:  1. Decision-Makers & Business Managers  2. BI Providers & IT Managers.  3.Policymakers.  This structure ensures that the implications are contextualized and practical, offering clear next steps for different stakeholders operating in an environment such as Yemen. | Section 6 (Discussion); Section 7.2 (Practice Implications) pages 17-22 |
| Reviewer #2 – 6: Many words are unclear, technical jargon is utilized without explanation, and tables and figures lack captions and narrative coherence. | **-Language and Jargon:** We have reviewed the entire text to simplify unclear language and define technical terms upon their first use (e.g., "R-SWARA," "TOEP framework"). The manuscript has also been proofread to enhance its overall fluency and academic tone.  **-Tables and Figures:** All tables and figures now have clear, descriptive captions that explain their purpose and content. | Entire manuscript |

We believe these revisions have substantially strengthened the manuscript's theoretical contribution, methodological rigor, and practical relevance. The responses address all concerns raised while maintaining the study's unique focus on Yemen's emerging market context.

Sincerely,
Amira Al-Adimi, Mokhtar Ghilan, Walid Yousef, and Abdullatif Ghallab
