# Supplementary material for: Critical success factors influencing business intelligence adoption: Evidence from Yemen
Source: PLoS One. 2026 Feb 25;21(2):e0343217. doi: 10.1371/journal.pone.0343217 (PMC12935231; doi:10.1371/journal.pone.0343217)
Supplement: Attachment — Submitted filename: Response_to_Reviewers_PONE.docx [file pone.0343217.s005.docx]

# Response to Reviewers

**Manuscript ID:** PONE-D-25-33603R1
**Title:** Critical Success Factors Influencing Business Intelligence Adoption: Evidence from Yemen
We sincerely thank the Academic Editor and the reviewers for their thoughtful comments and constructive feedback. Below we provide a detailed, point-by-point response in table format. Revisions are tracked in the file 'Revised Manuscript with Track Changes' and a clean version has also been uploaded.

| Reviewer Comment | Author Response | Location of Revision |
| --- | --- | --- |
| Reviewer #2: The theoretical contribution, albeit lucid, is somewhat constrained to a synthesis of previous models rather than representing a wholly novel theoretical advancement. | We thank the reviewer for this observation. We have revised the **(i) 'Theoretical Framework' section** to explicitly clarify the novelty of our theoretical advancement. While our model (TOEP) is a synthesis, we argue that it represents a systematic resolution of a reciprocal theoretical gap that neither parent model could address independently.  Specifically, the TOE framework, while holistic, lacks the 'procedural granularity' required for BI’s unique implementation lifecycle (as noted in Section 2.2). Conversely, the Yeoh & Koronios model, while process-centric, suffers from 'environmental blindness' by neglecting external pressures like competitive and regulatory factors (as noted in Section 2.3).  The novelty of the TOEP framework lies in its ability to reconcile these two perspectives into a single, unified lens specifically adapted for volatile, emerging contexts like Yemen. In such environments, the interplay between external instability (Environment) and internal change management (Process) is critical. This 'Contextual Theory Building' provides a more nuanced explanatory power than either model alone, thus offering a refined theoretical tool for future BI research.  And revised the **(ii) Theoretical Implications section** to emphasize that our contribution represents 'Contextual Theory Building'. We argue that the novelty lies in redefining the relationship between external volatility (Environment) and internal dynamics (Process). We have highlighted that in emerging and conflict-affected contexts like Yemen, this 'Process-centric' synthesis is a necessary theoretical advancement to move beyond generic IT adoption models, offering a refined analytical tool previously uncaptured in consolidated literature. | **(i)Theoretical Framework**: Paragraph 5, Lines 214-218.  **(ii) Theoretical Implications**: Paragraph 2 Lines 745-748. |
| Reviewer #2: Research would be more robust if it demonstrated the empirical superiority of the TOEP framework or its enhanced explanatory capacity relative to its foundational models. | To demonstrate the enhanced explanatory capacity of the **TOEP framework**, we have updated the **Discussion section** to show how the empirical results (specifically the high ranking of 'Environmental' and 'Process' factors) justifies the integrated approach. Without this synthesis, the critical influence of "Competitive Pressure" (Environment) and "Change Management" (Process) in the Yemeni context would have remained theoretically unexplained by either parent model alone. | **Discussion**: Paragraph 3, 4, 5 Lines 635-647. |
| Reviewer #2: Furthermore, although the rationale for using a cohort of twelve experts adheres to MCDM standards, the limited sample size constrains the external validity of the results. Gathering triangle data sources, such case studies or surveys, might enhance the generalizability of conclusions for further study. | We appreciate the reviewer’s observation regarding the sample size. We have addressed this by: **(i) Adding a detailed justification in the data collection** section confirming that a panel of 12 experts is not only consistent with R-SWARA and MCDM literature but is often considered optimal for maintaining the quality and depth of expert judgment.  **(ii) Including this as a 'Limitation'**, where we now suggest that future research could employ large-scale surveys or case studies to further validate the TOEP framework across other emerging or conflict-affected economies. | **(i) Data collection:**  Paragraph 2  Lines 516-519.    **(ii)Limitation and Future Work:**  Paragraph 2  Lines 849-853. |
| Reviewer #2: The methods section, although thorough, may be rather technical for those unacquainted with decision-making models; reducing the mathematical presentation or include a graphic summary would enhance accessibility. | Following the reviewer’s excellent suggestion, we have added **Fig 4**, which provides a **graphic summary** of the R-SWARA steps. This flowchart guides the reader through the logical progression from expert ranking to final weight derivation, making the process more accessible to non-technical readers. Furthermore, the detailed mathematical proofs and intermediate calculation tables (Steps 3-7) have been moved to **S1 Appendix** to improve the flow of the main manuscript. | **Research methodology, R-SWARA method:**  the Figure is added at the end of the section. |
| Reviewer #3: While the manuscript has improved, several sections especially in the literature review and methodology would benefit from an additional language polish to ensure smoother flow and reduce repetition. | We thank the reviewer for this suggestion. The entire manuscript has undergone a comprehensive language polishing and proofreading process. We have reviewed the manuscript to improve the narrative flow, eliminate redundant technical terms. | **The whole manuscript** |
| Reviewer #3: The link between each CSF ranking and its implications in the Yemeni context could be strengthened further. Some interpretations remain descriptive rather than analytical. | We appreciate this valuable feedback. We have significantly revised the implication section deepen the analysis of our findings within the Yemeni context. For instance, we now explicitly discuss how the prioritized factors (such as data quality and management support) are directly influenced by the current economic and operational challenges in Yemen. This shift from descriptive to analytical interpretation provides more practical insights for stakeholders in the region. |  |
| Reviewer #3: Ensure consistent referencing style throughout the manuscript. | We have meticulously reviewed the reference list to ensure full compliance with PLOS ONE’s formatting guidelines. | **The whole manuscript** |
| Reviewer #3: Check table numbering; ensure alignment with the narrative. | All figures/tables have been re-verified, re-numbered, and uploaded as high-resolution files. | **The whole manuscript** |
| Reviewer #3: Some technical terms should be defined once and not repeated excessively. | We appreciate this suggestion to improve the manuscript's readability. We have conducted a thorough review of the text to ensure that technical terms (such as 'Rough SWARA', 'Information-sharing culture', and 'TOEP framework') are defined clearly upon their first mention and used consistently thereafter. We have removed redundant definitions and excessive repetitions. | **The whole manuscript** |
| Reviewer #4: In general, please note that proofreading the paper may be beneficial. | We sincerely appreciate this suggestion. The entire manuscript has undergone a comprehensive proofreading and language editing process. We have corrected grammatical errors, improved sentence structure, and ensured that technical terminology is used consistently and accurately throughout the text. Special attention was given to the 'Methods' and 'Results' sections to ensure clarity and professional academic flow. | **The whole manuscript** |
| Reviewer #4: Introduction is poorly written. The flow of writing is missing throughout this section. Some critical shortfalls of this section are: (i) Research gap is not clear. Try to write the research gap more clearly and specifically. (i) Add relevant and most current years references. (iii) The novelty of the current study is not included in this section. Explain how this work is different from other works done in this field; that is the uniqueness of this study. | The Introduction has been **entirely rewritten** to address these concerns. We have: (i) specifically defined the research gap regarding BI in conflict-affected environments.  (ii) clearly articulated the uniqueness of the TOEP framework. And the R_SWARA employment for CSF prioritization. | (i) introduction: Paragraph 2, 3, 4.  Lines 35-52.  (ii) introduction:  Paragraph 5,6,7.  Lines 53-81. |
| Reviewer #4: Overlap between TOE and Yeoh & Koronios: The integration of TOE and Yeoh & Koronios is not fully justified. Both models already share organizational and technological dimensions. The added “Process” dimension could be argued as a subset of “Organization” in TOE. A clearer philosophical and theoretical justification for the synthesis is needed. | We appreciate the reviewer’s request for a deeper theoretical justification. We have addressed this by refining the manuscript in two key locations to clarify why 'Process' is treated as an independent dimension:   1. **First, in the Theoretical Development section:** We have integrated a detailed rationale based on the work of **Yeoh and Koronios (2010)**. We clarified that unlike static organizational traits, 'Process' represents the **dynamic, evolutionary journey** of BI implementation. We explicitly added that BI requires a dedicated focus on **incremental delivery** and **procedural methodology** to maintain strategic alignment—factors that are distinct from general organizational structure or technical readiness. 2. **Second, in the Theoretical Implications section:** We have highlighted the significance of isolating this dimension as a **theoretical contribution**. We clarified that by separating 'Process' from 'Organization', the **TOEP framework** provides the necessary **theoretical granularity** to analyze how structured workflows and change management act as the primary drivers of success in emerging and unstable environments like Yemen. This distinction allows for a more precise understanding of how 'active' implementation steps can overcome 'static' organizational barriers. 3. **Third, in the Practical Implication section:** We have explicitly highlighted the **Process dimension** in the Practical Implications section. We argue that the effectiveness of BI is not merely technical but procedural, as it ultimately depends on the willingness of the workforce to integrate these tools into their daily workflows. This justifies 'Process' as a vital, independent pillar in our **TOEP framework** | **1. Theoretical Framework**: Paragraph 4, Lines 200-213.  **2. Theoretical Implication:**  Paragraph 1, Lines 748-752.  **3. Practical implication,** **Guidance for Decision-Makers and Business Managers**: **the end of Paragraph 2,** Lines 790-792. |
| Reviewer #4: (1) The authors describe consent but do not name an Institutional Review Board (IRB) or provide an approval number. This is a mandatory requirement for PLOS ONE and must be added. | (1) We appreciate the reviewer’s important comment regarding ethical oversight. We have updated the manuscript to include the formal ethical approval details. This study was conducted in accordance with the ethical standards of the relevant academic authorities in Yemen. Formal ethical approval was obtained from the Faculty of Computing and Information Technology at Sana’a University (Approval No: 3002).  Furthermore, we have strictly adhered to the following ethical protocols: (i) Participants were informed that their involvement was voluntary and they could withdraw at any stage; (ii) All data were fully anonymized to ensure confidentiality. We have now added a dedicated 'Ethical Considerations' section to the revised manuscript and provided the official approval letter along with its English translation in **S3 Appendix** for further verification. | **(1) Research Methodology:** Ethical Considerations,  Lines 533-537.  And  **Supporting information:** S3**.** |
| Reviewer #4: The response mentions Appendix 1 with detailed tables, but it is not included in the provided PDF excerpt. The journal must ensure the appendix is submitted and accessible. | We apologize for the technical issue in the previous submission where the appendix was not visible. Following the **PLOS ONE Supporting Information guidelines**, we have now uploaded the detailed mathematical tables as a separate file labeled **'S1 Appendix'**. This file includes the step-by-step R-SWARA calculations and the expert scoring matrix. References to this supporting file have been embedded in the revised manuscript. | **Supporting information:** S1. |
| Reviewer #4: While expert judgment is central to R-SWARA, no measure of agreement among experts (e.g., Kendall’s W) is reported. This is a methodological weakness that should be acknowledged or addressed. | We sincerely thank the reviewer for this insightful observation. We agree that formal consensus measures like Kendall’s W are valuable in expert-based studies.  In this research, we utilized **Rough Set Theory** specifically because it is mathematically designed to handle uncertainty and diverse perspectives by defining lower and upper approximations. This allows for a robust 'interval of consensus' that captures expert subjectivity more effectively than traditional statistical tests in small, specialized panels.  However, to address your concern and enhance transparency, we have implemented the following:   1. **Methodological Justification:** We added a paragraph in the **Research** **Methodology** (R-SWARA method) section, explaining why Rough Set Theory was chosen as a robust alternative for managing expert divergence. 2. **Acknowledgment of Limitation:** We have explicitly added this point to the **Limitations and Future Research** section, noting that the absence of a formal consensus measure is a limitation and suggests the integration of such measures in future studies.   We believe this acknowledgment strengthens the manuscript and provides a clearer direction for future research. | **Research** **Methodology:** R-SWARA method) section**,**  Lines 435-438.  **And**  **Limitation:**  Lines 849-853. |
| Reviewer #4: The panel includes humanitarian sector experts. The authors should briefly justify why humanitarian experts are relevant to a business intelligence adoption study in Yemen. | We thank the reviewer for this insightful query. In the unique and challenging context of Yemen, the humanitarian sector is not merely a non-profit segment but a central pillar of the national economy and the data-management landscape. Due to the prolonged crisis, these organizations were among the earliest pioneers in Yemen to adopt and implement advanced Business Intelligence (BI) systems. This strategic adoption was necessitated by the critical need to manage and coordinate humanitarian aid for millions of beneficiaries whose numbers have surged due to the ongoing conflict and deteriorating socio-economic conditions. Handling such an immense scale of beneficiary data across diverse programs requires sophisticated BI tools to ensure precision, optimize logistics, and maintain the high levels of transparency demanded by international donors. Their early adoption and extensive experience in managing large-scale, high-stakes data under extreme environmental volatility provide a rich and mature perspective. Therefore, including experts from this sector was essential to capture a comprehensive view of BI implementation challenges and success factors in Yemen’s unique operational landscape. |  |
| Reviewer #4: In some places, “information culture” is used, elsewhere “information-sharing culture.” Standardize terms throughout. | We thank the reviewer for this observation. We have standardized the terminology throughout the entire manuscript. The term **'Information sharing culture'** is now used consistently. | **The whole manuscript** |
| Reviewer #4: Some figures and tables are referenced in the text but are not included in the submitted excerpt (e.g., Fig 1, Fig 2). Ensure all are present and correctly numbered. | We apologize for any confusion caused by the visibility of the figures in the previous excerpt. We would like to clarify that **Fig 1** and **Fig 2** were included in the original submission and appropriately referenced within the text.  However, to ensure full compliance and accessibility, we have re-uploaded all figures as high-resolution files according to the journal’s technical specifications. We have also carefully checked the numbering and placement of all tables and figures to ensure they are correctly integrated and visible in the generated PDF for the reviewers. | **The whole manuscript** |

We believe these revisions have substantially strengthened the manuscript's theoretical contribution, methodological rigor, and practical relevance. The responses address all concerns raised while maintaining the study's unique focus on Yemen's emerging market context.

Sincerely,
Amira Al-Adimi, Mokhtar Ghilan, Walid Yousef, and Abdullatif Ghallab
